# ALS Yeast Models—Past Success Stories and New Opportunities

**DOI:** 10.3389/fnmol.2018.00394

**Published:** 2018-10-30

**Authors:** Sonja E. Di Gregorio, Martin L. Duennwald

**Affiliations:** Schulich School of Medicine and Dentistry, Pathology and Laboratory Medicine, Western University, London, ON, Canada

**Keywords:** ALS, yeast, protein misfolding, neurodegeneration, proteinopathy

## Abstract

In the past two decades, yeast models have delivered profound insights into basic mechanisms of protein misfolding and the dysfunction of key cellular pathways associated with amyotrophic lateral sclerosis (ALS). Expressing ALS-associated proteins, such as superoxide dismutase (SOD1), TAR DNA binding protein 43 (TDP-43) and Fused in sarcoma (FUS), in yeast recapitulates major hallmarks of ALS pathology, including protein aggregation, mislocalization and cellular toxicity. Results from yeast have consistently been recapitulated in other model systems and even specimens from human patients, thus providing evidence for the power and validity of ALS yeast models. Focusing on impaired ribonucleic acid (RNA) metabolism and protein misfolding and their cytotoxic consequences in ALS, we summarize exemplary discoveries that originated from work in yeast. We also propose previously unexplored experimental strategies to modernize ALS yeast models, which will help to decipher the basic pathomechanisms underlying ALS and thus, possibly contribute to finding a cure.

## ALS

Amyotrophic lateral sclerosis (ALS) is a heterogeneous neurodegenerative disease caused by loss of the upper motor neurons, i.e., neurons that extend from the cortex to the brain stem and the spinal cord and lower motor neurons, i.e., neurons that connect the brainstem or spinal cord to muscle (Hardiman et al., 2017). Progressive loss of these neuron populations can manifest in two distinct early ALS symptoms: patients diagnosed with spinal-onset display a significant weakness of the limbs, whereas bulbar-onset leads to difficulty swallowing (dysphagia) and difficulty speaking (dysarthria; Hardiman et al., 2017). As the disease progresses, symptoms converge and death due to respiratory failure usually occurs within 3–5 years post diagnosis.

There is a substantial magnitude of heterogeneity of symptoms, variation of the age of onset and of disease progression in ALS. Comorbidity is observed with non-motor neuropathology in 50% of cases, with at least 13% of patients presenting concomitant behavioral variant frontotemporal dementia (FTD), which identifies ALS as a spectrum disorder rather than one single disease (Strong et al., 2017). ALS can also be grouped into either sporadic ALS (sALS), i.e., there is no family history, which accounts for ~90% of all ALS cases, or familial ALS (fALS), i.e., ALS is inherited within families, which accounts for the remaining ~10% of all ALS cases (Chen et al., 2013). The global incidence rate of the disease is approximately 1–2 new cases per 100,000 individuals with an overall prevalence averaging at 4–6 cases per 100,000 individuals (Chen et al., 2013). Despite considerable research efforts, the molecular mechanisms underpinning ALS remain mostly unknown and there is no cure. The substantial heterogeneity of ALS poses a significant problem in deciphering unifying ALS pathomechanisms. Yet basic cellular pathways, such as dysregulated ribonucleic acid (RNA) metabolism and protein misfolding and the associated toxicity appear to be highly common and key contributing factors to ALS pathogenesis.

## Impaired RNA Metabolism in ALS

RNA metabolism is a broad term encompassing the entire life cycle of all cellular RNAs, such as messenger RNA (mRNA), micro RNA (miRNA) and transfer RNA (tRNA). This includes RNA synthesis, modifications, folding and unfolding, processing and degradation, all of which are tightly regulated by multiple cellular pathways. RNA is synthesized from a DNA template by the process of transcription. Transcription is carried out in three steps of initiation, elongation and termination in a tightly controlled manner. Following termination, the synthesized RNA strand (hnRNA) must undergo post-transcriptional modifications before it can be translated at the ribosome in the case of mRNAs or processed into functional miRNAs or tRNAs. Finally, intervening introns are excised from the transcript to generate mature mRNAs (Krishnamurthy and Hampsey, 2009; Sainsbury et al., 2015). Modified mRNAs are then transported out of the nucleus and into the cytoplasm by a set of protein factors (Rodriguez et al., 2004). These messenger ribonucleoproteins diffuse through the nuclear pore complex and the protein factors are gradually removed to prepare the transcript for translation into protein by the ribosome. All RNAs can be degraded at any stage of their life cycle, allowing for dynamic regulation in the cell.

Perturbed RNA metabolism, particularly mRNA metabolism, plays a crucial role in the development of many neurodegenerative disorders, including ALS. Defects at all stages of the mRNA life cycle are prevalent in ALS and are mainly driven by disease-specific mutations in RNA binding proteins (RBPs). There are 10 RBPs with known ALS mutations in their encoding genes: ANG, EWSR1, Fused in sarcoma (FUS), hnRNPA1, hnRNPA2B1, RGNEF, SETX, TAF15, TIA-1 and TAR DNA binding protein 43 (TDP-43; Table 1). These mutations lead to a broad range of deficits in RNA metabolism, including impaired transcription of both mRNAs and miRNAs, post-transcriptional modifications and RNA editing. Many of the RBPs affected in ALS participate in the formation of stress granules (SGs) under cellular stress to halt non-essential translation and to sequester and preserve specific mRNAs.

**Table 1:** A list of the most common genes implicated in amyotrophic lateral sclerosis (ALS) and other neurodegenerative disorders. Known biological functions of each protein are listed.

Protein	RNA binding protein	Normal function	Disease	Reference
TDP-43	Yes	RNA metabolism	ALS (FTLD/ALS)	Sreedharan et al. (2008) and Kirby et al. (2010)
FUS	Yes	RNA metabolism	ALS (FTLD/ALS)	Kwiatkowski et al. (2009) and Vance et al. (2009)
SOD1	No	Oxidative stress	ALS	Rosen et al. (1993) and Andersen (2006)
C9orf72	Yes	RNA metabolism/RNA processing, nucleocytoplasmic transport	ALS, FTLD/ALS, FTD	DeJesus-Hernandez et al. (2011) and Renton et al. (2011)
Ataxin-2	No	Caspase activation, TDP-43 modification	ALS, PD, Ataxias	Elden et al. (2010)
Tau	No	Microtubule homeostasis	FTD, AD, Tauopathy	Lin et al. (2017)
OPTN	No	Autophagy	ALS	Maruyama et al. (2010)
PFN1	No	Cytoskeleton, actin polymerization	ALS	Wu et al. (2012)
hnRNPA1, hnRNPA2B1	Yes	RNA metabolism and transport	ALS, FTLD/ALS, FTD	Kim et al. (2013)
VAPB	No	Vesicle trafficking	ALS	Nishimura et al. (2004, 2005)
VCP	No	Protein degradation	ALS, FTLD/ALS, FTD, MJD, HD, PD	Johnson et al. (2010)
SETX	Yes	DNA/RNA Helicase, RNA Metabolism	ALS	Chen et al. (2004)
DCTN1	No	Axonal transport	ALS, FTLD/ALS	Münch et al. (2004); Münch et al. (2005)
NEFH	No	Neurofilament component	ALS	Figlewicz et al. (1994)
ALS2	No	Rho GEF, Vesicle transport	Juvenile ALS	Hadano et al. (2001) and Yang et al. (2001)
CHMP2B	No	Vesicle transport	ALS, FTD	Parkinson et al. (2006) and Cox et al. (2010)
ANG	Yes	RNA metabolism	ALS, FTLD/ALS	Greenway et al. (2004); Greenway et al. (2006)
UBQLN2	No	Targeting misfolded proteins to proteasome, autophagy	ALS, FTLD/ALS	Deng et al. (2011)
SQSTM1	No	Autophagy, NFkB activator	ALS, FTLD/ALS	Fecto et al. (2011)
TUBA4A	No	Microtubule component	ALS	Smith et al. (2014)
7TBK1	No	NFkB activator, vesicle transport, autophagy	ALS	Cirulli et al. (2015) and Freischmidt et al. (2015)
C21orf2	No	Cilia formation, DNA repair	ALS	van Rheenen et al. (2016)
NEK1	No	Cilia formation, DNA repair	ALS	Kenna et al. (2016)
CHCHD10	No	Oxidative Phosphorylation	ALS, FTLD/ALS, FTD	Bannwarth et al. (2014) and Johnson et al. (2014)
TAF 15	Yes	RNA Metabolism	ALS	Couthouis et al. (2011)

For example, TAF15 is a component of the TFIID complex that is essential for RNA polymerase II transcription (Bertolotti et al., 1996; Kwon et al., 2013). Mutations in the gene encoding TAF15 have been uncovered in ALS patients but are not present in unaffected controls (Couthouis et al., 2011; Ticozzi et al., 2011). An overarching theme amongst ALS RBPs is their structural similarities. TAF15 shares sequence and domain homology with both TDP-43 and FUS and all three proteins may at least partially overlap in function. Both FUS and TAF15 belong to the FET family of heterogeneous nuclear ribonucleoproteins (hnRNPs) and like TDP-43 and FUS, TAF15 functions in alternative splicing and transcription. Furthermore, the majority of TAF15 ALS mutations are located within the glycine-rich region or prion-like-domain at the C-terminus of the protein, with similar ALS-associated mutations found in TDP-43 and FUS. Finally, TAF15 also mislocalizes from the nucleus into the cytoplasm and is found in cytoplasmic inclusions, a common pathological hallmark in ALS proteinopathy, which is also well-established for TDP-43 and FUS.

## Protein Misfolding in ALS

Protein misfolding describes the conversion of proteins from their normal, mostly soluble and functional three-dimensional conformations into aberrant, often insoluble, non-functional conformations (Soto, 2003; Soto and Estrada, 2008; Sweeney et al., 2017). This can result in a toxic gain-of-function or loss-of-function of the disease gene or protein, or a combination of both, which cause neurodegeneration. Most neurodegenerative diseases, such as Alzheimer’s disease, Parkinson’s disease, Huntington’s disease and ALS are protein misfolding diseases. Genetic mutations can cause a protein to misfold, e.g., the misfolded huntingtin protein in Huntington’s disease. However, in Parkinson’s, Alzheimer’s and ALS, most cases cannot be associated with any known mutations. Environmental insults, such as changes in pH and exposure to toxic chemicals or oxidative stress, can lead to protein misfolding that may contribute to neurodegeneration. Finally, the highest risk factor for most neurodegenerative diseases is advanced age, indicating that the physiological changes associated with aging contribute to disease-related protein misfolding.

Like most neurodegenerative diseases, ALS is characterized by protein misfolding and protein aggregation in affected neurons (Sweeney et al., 2017). These misfolded proteins and aggregates, containing proteins, such as TDP-43, FUS, C9orf72, superoxide dismutase (SOD1) and many others, are well-established pathological hallmarks of ALS (Okamoto et al., 1991; Watanabe et al., 2001; Arai et al., 2006; Neumann et al., 2006; Mackenzie et al., 2007; Kwiatkowski et al., 2009; Vance et al., 2009; Al-Sarraj et al., 2011). Yet it remains unclear how these misfolded and aggregated proteins execute neurotoxic functions and contribute to the ALS-specific pattern of neurodegeneration. Like many other neurodegenerative diseases, ALS is characterized by the highly selective demise of specific neurons, mostly motor neurons, while other neurons remain unaffected (Saxena and Caroni, 2011). This implies that the affected neurons are unable to avert the toxic consequences of ALS-specific protein misfolding and aggregation and that the defensive mechanisms that normally combat protein misfolding are ineffective. By contrast, unaffected neurons seem to be able to avert the toxic consequences of protein misfolding or even protein misfolding itself.

The newly discovered liquid-to-solid phase transition of the ALS protein FUS has added an additional layer of complexity to the well-known aspects of protein misfolding (Murakami et al., 2015; Patel et al., 2015; Monahan et al., 2017; Qamar et al., 2018). Phase transition begins with the single monomer of an intrinsically disordered protein harboring a prion-like domain (PrLD; St. George-Hyslop et al., 2018). The monomers exist in liquid-liquid phase separation under physiological conditions as spherical droplet structures. These structures are an example of a non-membrane bound compartment distinguished from the cytoplasm or the nucleoplasm by their condensed liquid state. When multiple FUS droplets come into contact, they quickly fuse and arrange into a larger droplet. This is governed by relatively weak, transient and homotypic interactions between the aggregation-prone domains of the protein. Patel et al. (2015) have shown that larger droplet formations of multiple droplets carry the potential to undergo aberrant liquid-solid phase transition which results in the formation of solid, fibrous aggregates. ALS-associated mutations in FUS can expedite this process (Patel et al., 2015).

Neurodegeneration is closely linked to prion-like conversion of properly folded to misfolded proteins and the spreading of neuropathology from cell to cell (Scheckel and Aguzzi, 2018). The concept of prion and prion-like-behavior is rooted in earlier work on the mammalian prion PrP and yeast prions. Domains of low-sequence complexity form compartments unbound by membranes, similar to the liquid-solid phase transition of FUS (Brangwynne et al., 2009; Kato et al., 2012; Patel et al., 2015). Yeast prions contain low complexity domains that readily transition into solid, aggregate fibers rather than a liquid state (Liebman and Chernoff, 2012). Thus, comparable domains in proteins were coined “prion-like.” Low complexity PrLDs are common in RNA/DNA binding proteins, such as FUS and TAR DNA binding protein 43 (TDP-43), and many other known ALS proteins (Gitler and Shorter, 2011). It is plausible that prion-like conversion and seeding mechanisms of protein misfolding is central to the spreading of ALS pathology, e.g., from neuron to neuron.

## Cell Stress Responses—From Humans to YEAST

There are three major cellular stress response programs regulating protein quality control that protect cells from the toxic effects of protein misfolding: (1) the heat shock response (HSR); (2) the antioxidant response (AR) both of which act in the cytoplasm; and (3) the unfolded protein response (UPR) which acts in the endoplasmic reticulum and secretory pathway.

The HSR is a highly conserved pathway activated to prevent or repair the damages caused by heat and other stressors (Richter et al., 2010). In humans and yeast the HSR is regulated primarily by the transcription factor heat shock factor 1 (Hsf1), which is responsible for transient induction of the expression of heat shock stress proteins (Hsps) and molecular chaperones (Wu, 1984; Wu et al., 1986; Richter et al., 2010). Oxidative stress contributes to the pathogenesis of neurodegenerative diseases (Kim et al., 2015). Oxidative stress is defined as the imbalance between reactive oxygen species (ROS) and reactive nitrogen species (NOS) and the defensive cellular AR (Camhi et al., 1995). Prolonged damage to the cell can be inflicted by ROS on nucleic acids, proteins and membrane lipids. Oxidative stress is a major contributor to many neurodegenerative diseases. The UPR collectively describes multiple pathways dedicated to maintaining proteostasis in the ER and secretory pathway (Hetz and Papa, 2018). These processes are constitutively active at basal levels, however, in the presence of accumulated misfolded proteins in the ER, quality control mechanisms become overwhelmed leading to ER stress. This leads to rapid activation of the UPR, via three central signal proteins, the protein kinase-like ER kinase (PERK), activating transcription factor 6 (ATF6) and inositol-requiring enzyme-1 (Ire1a), which results in strong induction of the expression of proteins involved in protein folding, ER-associated degradation (ERAD), vesicular trafficking, ER redox control, amino acid metabolism, lipid synthesis and autophagy. Only the Ire1 UPR signaling pathway is conserved from yeast to human cells.

Previous work in cultured cells and transgenic mice, strongly indicates that all three stress response pathways are malfunctioning and contribute to ALS pathogenesis (Atkin et al., 2008; Wang et al., 2011). Yet what role the dysregulation of these stress response pathways plays in human ALS neurons, particularly in those neurons strongly affected in ALS, has not yet been examined in a systematic manner.

## Yeast Models—Opportunities and Limitations

The contributions from studies in yeast to our understanding of basic mechanisms underlying ALS and identifying key proteins has been substantial. *Saccharomyces* *cerevisiae* (yeast) is a single-celled organism and was the first eukaryote to have its genome fully sequenced (Goffeau et al., 1996). Nearly a third of yeast genes have a direct human ortholog and more than two thirds have significant homology with human genes (Laurent et al., 2016). Approximately 500 genes implicated in human disease have a direct ortholog in yeast, implicating the tractability of yeast as a model to study human disease (Kryndushkin and Shewmaker, 2011). The strengths of the yeast model arise from our considerable understanding of basic cell biology, genetics and biochemistry.

A multitude of genetic, microscopic and biochemical tools have been developed, such as high-throughput screens, which are not yet possible to this the same extent in any other model eukaryotic organism. These screens are highly versatile and allow the detection of novel genetic and protein-protein interactions. Over-expression and deletion libraries of the entire yeast genome allow identifying and characterizing modifiers of a target misfolded protein. Such studies have elucidated previously unexplored mechanisms in many neurodegenerative disorders, including ALS (Yeger-Lotem et al., 2009; Elden et al., 2010; Khurana and Lindquist, 2010; Treusch et al., 2011; Kim et al., 2014).

**Figure 1:**
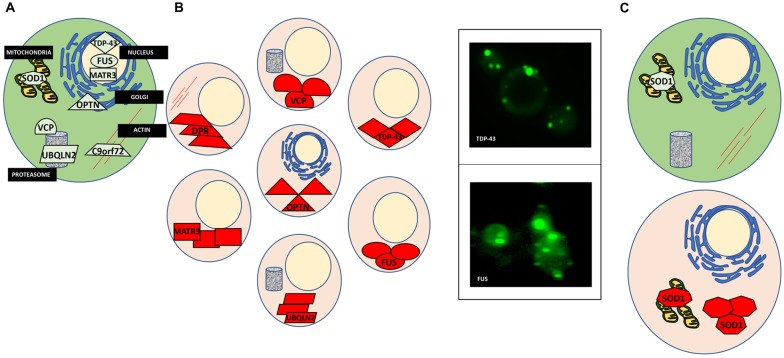
Protein misfolding in amyotrophic lateral sclerosis (ALS). **(A)** A normal cell depicting natively folded proteins in their proper location compared to ALS cells where proteins are found mislocalized and aggregated. **(B)** TAR DNA binding protein 43 (TDP-43) is mislocalized from the nucleus and aggregated within the cytosol (top left). Yeast models of ALS recapitulate these features of TDP-43 proteinopathy (top right). GFP-tagged TDP-43 wild-type expressed in yeast is found in cytoplasmic inclusions throughout the cell. Fused in sarcoma (FUS) proteinopathy is similar to that of TDP-43 (bottom left and right). **(C)** Superoxide dismutase (SOD1) is localized to the mitochondria and throughout the cytosol. In ALS, misfolded SOD1 is found aggregated at these locations.

**Figure 2:**
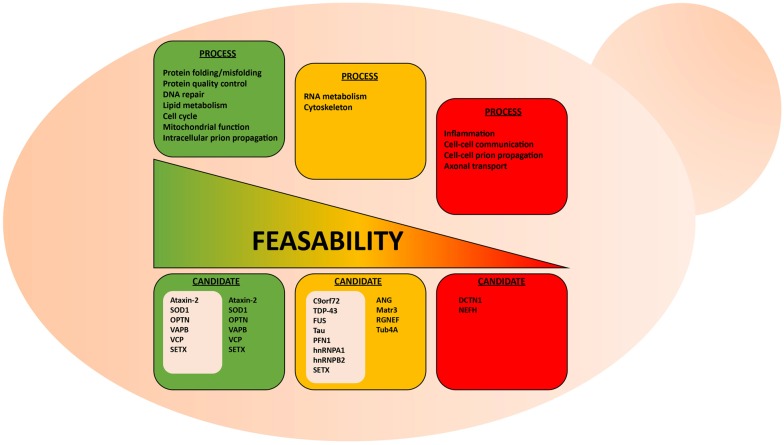
Suitability of the yeast model system to study various aspects of ALS. Highly conserved biological processes, such as protein misfolding and protein quality control, are better suited for studies in yeast. Here, examples of conserved and non-conserved processes are listed for candidate ALS proteins already studied in yeast. These ALS proteins are grouped in gray-colored boxes.

The cellular processes that involve protein misfolding and in turn the cellular response to protein misfolding, i.e., cellular stress response pathways, are highly conserved between humans and yeast (Winderickx et al., 2008). As a consequence, many yeast models of protein misfolding diseases recapitulate the general patterns of mislocalization, aggregation and cellular quality control mechanisms (Figure 1; Winderickx et al., 2008). Additionally, cellular quality control mechanisms, including the HSR and the UPR, are heavily conserved. While the focus of this review article is on impaired RNA metabolism and protein misfolding, yeast models also recapitulate many other essential mechanisms of eukaryotic biology. Cell cycle regulation, organelle function, and DNA metabolism are all examples of highly tractable process that can be aptly studied in yeast (Figure 2).

Using yeast as a living test-tube undoubtedly has a firm place in our experimental repertoire to explore neurodegenerative diseases, yet some caveats should be considered when assessing the suitability of yeast models. For instance, certain cellular mechanisms, such as cytoskeletal regulation and certain aspects of RNA metabolism, are not highly conserved between yeast and human neurons (Lemmens et al., 2010; Kevenaar and Hoogenraad, 2015). The simplification of such systems can therefore be problematic if not properly considered. For example, yeast do not contain neurofilaments, which are heteropolymers that form the neuronal cytoskeleton along with microfilaments and tubulin. While neurofilaments seem to contribute to ALS pathogenesis (Mendonça et al., 2005; Petzold, 2005; Gnanapavan et al., 2013), it might thus be problematic to study neurofilament-associated aspects of cytoskeleton disorganization in ALS yeast models. Similarly, certain aspects of RNA metabolism, i.e., RNA transport, degradation and translation, differ in yeast and mammalian cells (Lemmens et al., 2010). Only a small number of yeast genes possess introns and there are notable differences in the intron region of pre-mRNA that are essential for splicing between yeast and human cells. Also, yeast does not possess the miRNA processing machinery characteristic of human cells. Considering the substantial amount of RNA metabolism regulators implicated in ALS (Tables 1, 2), it is important to understand these limitations when using yeast models. Yet, many of the core aspects of RNA metabolism, particularly mRNA processing, are similar between yeast and humans and, thus, some ALS-related RNA mechanisms can most likely be evaluated in yeast models.

**Table 2:** Published ALS yeast models and their characteristics.

Human ALS protein (wild-type and mutants)	Toxicity	Aggregation	Reference
TDP-43 G294A, Q331K, M337V, Q343R, N345K, R361S, N390D	Yes	Yes	Johnson et al. (2009), Armakola et al. (2011), Braun et al. (2011), Kryndushkin and Shewmaker (2011), Sun et al. (2011), Jackrel et al. (2014), Liu et al. (2017) and Leibiger et al. (2018)
FUS R524S, P525L	Yes	Yes	Fushimi et al. (2011), Ju et al. (2011), Kryndushkin and Shewmaker (2011), Kryndushkin et al. (2011), Sun et al. (2011), Daigle et al. (2013) and Jackrel et al. (2014)
SOD1 A3V, G36R, H47Q, G92A, S133N	No	No	Nishida et al. (1994), Rabizadeh et al. (1995), Corson et al. (1998), Gunther et al. (2004), Bastow et al. (2011) and Bastow et al. (2016)
C9orf72 (GA)50, (GR)100, (PA)50, (PR)50	Yes	Not assessed	Jovičić et al. (2015) and Chai and Gitler (2018)
Ataxin-2 Q22, Q79	No	Not assessed	Ralser et al. (2005), Nonhoff et al. (2007), Elden et al. (2010) and Bonini and Gitler (2011)
OPTN E50K, E478G	Yes	Yes	Kryndushkin et al. (2012)
PFN1 C71G, T109M, M114T, E117G, G118V, R136W, H120E	No	No assessed	Figley et al. (2014)
hnRNPA1, hnRNPA2B1 hnRNPA1 D262V, hnRNPA1 D262N, hnRNPA2B1 D290V	Yes	Yes	Kim et al. (2013)
VAPB P56S	Wild-type—Yes P56S—not assessed	Yes	Suzuki et al. (2009), Nakamichi et al. (2011)
VCP R155C, A232E, T761E, K524A	No	Wild-type, T761E, K524A—No R155C and A232E—Yes	Takata et al. (2012)
SETX	No	No	Richard et al. (2013), Bennett and La Spada (2018)
ANG	Yes	Yes	Jo et al. (2017)
TAF 15	Yes	Yes	Couthouis et al. (2011)
UBQLN2 (essential domain only)	No	Not assessed	Gilpin et al. (2015)

*Wild-type and ALS-associated mutant proteins that have been expressed in yeast are listed and categorized based on toxicity and aggregation phenotypes. Toxicity refers to growth defects of yeast cells expressing ALS proteins. Aggregation refers to fluorescent microscopically assessed inclusions or foci. Note, that even though there is a strong correlated between aggregation and toxicity of ALS proteins in yeast, this does not equate with a causative relation*.

Clearly, the suitability of the yeast model depends on what research question is explored (Figure 2). It seems obvious that there are certain questions that cannot be answered within this single-celled organism, e.g., macro-physiological or tissue-specific processes, such as inflammation and prion-like cell to cell spreading from cell to cell. Also, exploring highly specialized neuronal functions, such as synaptic communication and axonal transport in ALS make effective studies difficult in yeast models.

## Yeast Models of ALS

We count more than 13 published ALS yeast models, i.e., yeast expressing different ALS-associated protein (Table 2, Van Damme et al., 2017). Many of these are not yet developed to the extent of the TDP-43 and FUS models, however, important commonalities between these models are emerging. Figure 1 illustrates the aggregation and mislocalization of ALS proteins, a hallmark of pathology that is consistently recapitulated in yeast. It is noteworthy that even though in all listed examples in Tables 1, 2 lists all of these ALS yeast models, the associated proteins, and major findings. A major similarity between all these proteins, regardless of their diverse biological function, is protein misfolding and many of them have RNA binding function.

Of the 10 known RBPs involved in ALS, nine of them have been successfully modeled in yeast. ANG is involved in the processing of ribosomal RNA and has been shown to act as stress-activated RNase that promotes SG assembly by cleaving tRNA and inhibiting translation (Shapiro et al., 1986; Harper and Vallee, 1989). The yeast proteome does not contain any ANG homolog and ANG expression is highly toxic in yeast, both of which are common features of many ALS yeast models (e.g., TDP-43 and FUS but not SOD1, which has a yeast homolog). Yeast high-throughput screens using human libraries identified genetic modifiers of ANG toxicity (Jo et al., 2017), i.e., potent suppressors of ANG toxicity. Deletion of a subset of these suppressor genes also attenuated protein aggregation in these models. Four of the suppressors uncovered novel interactions between ANG and the ALS protein OPTN.

Another RBP that has been studied in yeast is SETX, an RNA/DNA helicase. SETX has been shown to function as an RNA Polymerase II transcription terminator by resolving R-loops and allowing the 5′-3′exoribonuclease Xrn2 to degrade the RNA transcript following extended pausing at G-rich sites (Skourti-Stathaki et al., 2011). Interactor screens in the yeast-2-hybrid system revealed an interaction between SETX and Rpr45, a component of the exosome complex important for RNA turnover and quality control (Richard et al., 2013). This interaction depends upon sumoylation of SETX. ALS mutants of SETX were also examined, however, it was found that ALS-associated mutations did not disrupt interaction with Rpr45. Following up on these findings, the authors demonstrated co-localization of SETX and Rpr45 in the nucleus in mammalian cell lines. This occurred as a response to induced DNA damage, suggesting a new role for the exosome is DNA repair that may have implications in ALS (Richard et al., 2013).

The most prominent ALS yeast model is the TDP-43. TDP-43 is a DNA/RBP involved in RNA metabolism and one of the most common genetic causes of ALS (Chen et al., 2013). Over 40 ALS mutations have been discovered in TDP-43 which accounts for 4%–5% of fALS and 2% of sALS. Notably, almost all identified mutations are missense mutations in the glycine-rich C-terminal region, also known as a PrLD. This region is important for protein-protein interactions and likely a central contributor to TDP-43 misfolding (Gitler and Shorter, 2011). TDP-43 is the most common component of hallmark ALS cytoplasmic inclusions independent of mutated forms of the protein (Mackenzie and Rademakers, 2008). Approximately 97% of ALS patients demonstrate TDP-43 proteinopathy, where the protein is found mislocalized, i.e., expelled from the nucleus and misfolded into aggregates in the cytoplasm, a phenomenon coined TDP-43 proteinopathy.

Yeast as a model of TDP-43 proteinopathy has proven quite useful, recapitulating the major characteristics of the misfolded protein in the disease (Johnson et al., 2009; Armakola et al., 2011; Kryndushkin and Shewmaker, 2011; Sun et al., 2011). When expressed in yeast, TDP-43 is found outside the nucleus in soluble aggregates in the cytosol (Figure 1B). Many of the ALS-associated mutants have also been modeled in yeast and compared to the wild-type TDP-43 protein. These studies revealed that ALS mutations increased the propensity of TDP-43 to aggregate and increased toxicity (Johnson et al., 2009; Armakola et al., 2011; Kryndushkin and Shewmaker, 2011; Sun et al., 2011). Additionally, TDP-43 is toxic in yeast in a dose-dependent manner, making it a highly suitable candidate for high-throughput screens to identify genes and proteins that modulate its toxicity. From these screens many previously undescribed genetic interactions of TDP-43 have been identified, the most significant being the modulation of TDP-43 toxicity by ATAXIN-2 (ATXN2), the polyQ protein mutated in spinocerebellar ataxia type 2 (SCA2). A study by Gitler and co-workers revealed that PBP1, the yeast homolog of ATXN2, is a potent enhancer of TDP-43 toxicity when overexpressed simultaneously in yeast (Elden et al., 2010). Concurrently, when expressed in strains genetically deleted for gene encoding Pbp1, TDP-43 toxicity was reduced. Also, the upregulation of Pbp1 increased the number of fluorescent foci of fluorescent protein-tagged TDP-43 in yeast. These critical findings established the importance of ATXN2 as a common contributor to ALS and provided yet another example of proteins misfolding across multiple neurodegenerative disorders, as ATXN2 can also contribute to Parkinson’s disease and mutations in ATXN2 cause SCA2 (Imbert et al., 1996; Pulst et al., 1996; Sanpei et al., 1996; Lorenzetti et al., 1997; Infante et al., 2004; Nanetti et al., 2009; Fischbeck and Pulst, 2011). Importantly, subsequent studies in human cell culture, fly and mouse models have confirmed these results from yeast (Bonini and Gitler, 2011).

Similarly, wild-type and ALS mutants of FUS, another misfolded RBP in ALS, have also been successfully studied in yeast. As with TDP-43, FUS is mislocalized from the nucleus and found aggregated in the cytoplasm in ALS post-mortem tissues (FUS proteinopathy) and mammalian cell models (Mackenzie et al., 2011; Shang and Huang, 2016; Sharma et al., 2016). This holds true in yeast, as FUS is found outside of the nucleus and sequestered into aggregates in the cytosol (Figure 1B; Fushimi et al., 2011; Ju et al., 2011; Kryndushkin and Shewmaker, 2011; Kryndushkin et al., 2011; Sun et al., 2011). Like TDP-43, FUS also contains a glycine-rich region and NLS where most ALS-associated mutations occur. Studies in yeast have helped delineate which domains contribute to protein misfolding, aggregation, and the formation of aberrant protein-protein interactions (Sun et al., 2011). In addition, studies in yeast revealed that FUS induces the formation of RNA granules and localizes there along with other components such as Pbp1. Furthermore, deletion of the RNA recognition motif in FUS did not alter aggregation, however, rescued toxicity in yeast, demonstrating that the ability of FUS to bind RNA is required for FUS toxicity, providing an fascinating example of the interplay between protein misfolding and RNA metabolism in ALS (Sun et al., 2011).

hnRNPs, A2B1 and A1, are additional examples of an RBPs with a PrLDs implicated in ALS (Kim et al., 2013). These proteins function in partnership with TDP-43 in pre-mRNA splicing, mRNA transport, transcript stability and translation regulation (Martinez et al., 2016). As with TDP-43 and FUS, the disease-causing mutations fall within the PrLD of each protein and are predicted to enhance aggregation propensity. As a result, they are recruited to SGs and cytoplasmic inclusions similar to other ALS RBPs (Martinez et al., 2016). Kim et al. (2013) characterized a yeast model expressing A2B1 wild-type and the D290V mutant and found that both variants are highly toxic and form fluorescent foci in yeast. HnRNPA1 wild-type and two mutants, D262V and D262N, were also characterized in yeast with similar phenotypes. They found that both hnRNPs demonstrated greater toxicity in yeast than either TDP-43 or FUS and their mutants. Unlike ALS mutants of TDP-43 and FUS, hnRNP toxicity was not increased in the mutants. Considering the prevalence of RBPs with PrLDs and the role these domains play in driving the development of ALS, there is a strong basis to conclude that both RNA metabolism and protein misfolding are strongly linked (Kim et al., 2013).

VAPB is involved with vesicular trafficking, a process known to be involved in ALS and many other neurodegenerative disorders (Suzuki et al., 2009; Nakamichi et al., 2011). Importantly, several proteins involved in autophagy and protein degradation, such as OPTN and VCP, have also been previously studied in yeast (Kryndushkin et al., 2012; Takata et al., 2012).

Mutations in the gene encoding Copper, Zink SOD1, a conserved cytosolic ROS scavenger, were the first identified genetic causes of fALS, and until the discovery of TDP-43 and FUS in 2006, SOD1 was the only known ALS gene. There is an extensive body of literature dedicated to the study of SOD1 wild-type and over 160 known ALS mutations (Rosen et al., 1993; Bunton-Stasyshyn et al., 2015). Unlike TDP-43 and FUS, SOD1 mutations are scattered throughout the entire protein and likely affect more than one of its biochemical properties and biological functions (Cleveland and Rothstein, 2001). SOD1 and a subset of ALS-linked mutations have been introduced into yeast (Tables 1, 2, Nishida et al., 1994; Gunther et al., 2004; Bastow et al., 2016). Intriguingly, neither the wild-type protein or any of the currently modeled ALS mutations in yeast demonstrate any severe growth defect (Nishida et al., 1994; Rabizadeh et al., 1995; Corson et al., 1998; Gunther et al., 2004; Bastow et al., 2011, 2016). Deletion of the yeast SOD1 homolog revealed that human SOD1 could fully complement the biological function of yeast SOD1 (Martins and English, 2014). This lead to the discovery that many of the mutated SOD1 proteins in ALS retain full enzymatic function (Bastow et al., 2016). There is, however, a marked propensity for the wild-type SOD1 protein and even more so for ALS-associated SOD1 mutants to selectively aggregate close to mitochondria where the protein may confer a toxic function that is not yet fully understood (Figure 1C; Vijayvergiya et al., 2005). The relationship between the ALS mutations and the apparent toxicity remains enigmatic and seems quite distinct from other ALS proteins studied in yeast. Also, experiments in yeast and other model systems demonstrated interference by mutant SOD1 with ER-Golgi transport. Unlike TDP-43 and FUS, where aggregation propensity correlates with growth defects, yeast models of SOD1 do not reveal toxicity in the presence of mitochondrial inclusions (Figure 1C; Nishida et al., 1994; Gunther et al., 2004; Bastow et al., 2011, 2016). All these results challenge a simple correlation between protein misfolding or aggregation and toxicity for SOD1 and its ALS-associated mutants. Traditionally, aggregation has been considered detrimental to the cell. Yet increasing evidence suggests that sequestering misfolded proteins can also be protective and facilitated by cellular protein quality control mechanisms, e.g., molecular chaperones (Chen et al., 2011; Takalo et al., 2013). It is also known that SOD1 localizes to intermembrane compartment of the mitochondria and exerts a protective function against ROS, thus cautioning the proper distinction between normal localization and aberrant aggregation (Chen et al., 2011; Fischer et al., 2011). Continued work in yeast and other model systems will further delineate the intricate relationships between SOD1 and its ALS-associated mutations and their toxicity, localization, misfolding and aggregation.

In 2011, the discovery of intronic, hexanucleotide repeats of the C9Orf72 gene revealed the most common cause of fALS-FTD (Renton et al., 2011; Freibaum and Taylor, 2017). The GGGGCC repeats lower expression of the C9Orf72 protein product and accumulation of repeat-containing RNA may sequester RBPs to confer a toxic gain of function. Importantly, unconventional translation of RNA containing the GGGGCC repeats produces aberrant dipeptide repeat (DPR) proteins that accumulate in motor neurons and may seed the early stages of the disease (Figure 1A; Freibaum and Taylor, 2017). There are five different DPRs: glycine–alanine (GA), glycine–arginine (GR), proline–alanine (PA), proline–arginine (PR) and glycine–proline (GP). All five DPRs have been modeled in yeast and other model systems. As in human cells and *Drosophila*, the GR and PR DPRs are toxic in yeast (Jovičić et al., 2015; Chai and Gitler, 2018). This phenotype was exploited by Gitler and colleagues to investigate the specific causes of toxicity in high throughput-enhancer and suppressor screens in yeast. These studies revealed 133 gene deletions that supressed the toxicity phenotype associated with the expression of GR_100_, a construct with 100 DPRs. Many of these modifiers are related to ribosome biogenesis. These deletions had not been identified in previous screens for genetic modifiers of other ALS proteins (e.g., FUS or TDP-43), suggesting a DPR-specific mechanism of toxicity (Jovičić et al., 2015; Chai and Gitler, 2018).

## The Future of ALS Studies in Yeast

Despite the considerable body of ALS yeast literature, we argue that there remains a largely untapped potential of this model system. Many of the complex mechanisms underlying ALS onset might be rooted in protein-protein interactions. Yeast is an excellent platform for the discovery of novel interactors but also for the characterization of such relationships. The Split-ubiquitin assay is one effective alternative to the classical yeast-two-hybrid system that can detect protein interactions without translocation to the nucleus and proteins bound to the cell membrane (Johnsson and Varshavsky, 1994; Müller and Johnsson, 2008). Many methods such as pull-down assays that detect protein-protein interactions are also highly suitable for studies in yeast with its well-described proteome (Xing et al., 2016). In addition, the interplay between different ALS-associated proteins and mutations remains poorly understood. Future protein-protein interaction and genetic studies in yeast present the ideal scenario to retrieve novel information of the basic biology of these interactions. For instance, it will be important to explore how the misfolding of one ALS protein, modulates the misfolding of another, for example, how TDP-43 misfolding modulates SOD1 misfolding and toxicity.

Studies on yeast prions have been tremendously successful in deciphering basic mechanisms underlying prion propagation and prion maintenance (Liebman and Chernoff, 2012). Yeast prions are self-perpetuating protein aggregates or conformers that confer a transmissible and heritable phenotype in a non-Mendelian inheritance pattern (Liebman and Chernoff, 2012). Considering what is already known about ALS proteins containing PrLDs (e.g., TDP-43 and FUS), and the newly proposed mechanism of phase transition of the FUS protein, similar investigations in yeast can probably enhance our understanding of protein misfolding in ALS. For example, very little is known about the events that precede the formation of hallmark inclusions in ALS that contain both FUS and TDP-43. Future studies in yeast could delineate the nature of recruitment of these proteins to inclusions.

Additionally, as we recently reviewed in detail, yeast is also a very suitable model to study the aspects of aging, which play an important role in most neurodegeneration, including ALS (Di Gregorio and Duennwald, 2018). In brief, there are two distinguished paradigms of yeast aging models: chronological and replicative aging. Chronological aging describes the length of time a yeast cell can remain viable and replicative aging describes the number of cellular divisions a mother cell can undergo before senescence. Yeast growth follows the classical “S”-curve stages of divisions, beginning with the lag phase, transitioning into the log phase, and finally the stationary phase (cells cease dividing). Yeast cells are “aged” following the diauxic shift that occurs toward the end of the log phase. Thus, “young” cells are still in lag and early log phase. These wild-type phases are defined by different metabolic profiles and rates of division. Importantly, aged yeast cells recapitulate many of the important aspects of mammalian cell counterparts and specifically, aged yeast more closely resemble neurons: older yeast cells undergo G1 cell cycle arrest, show increased ROS and autophagy, and metabolically switch to oxidative phosphorylation. All these are commonalities between aged yeast and neuronal cells. Thus, studying ALS-associated protein misfolding in aged yeast model may reveal how aging contributes to protein misfolding and the associated toxicity.

Yeast models can also serve to study the impact of different metabolic states, e.g., energy production by glycolysis compared to oxidative phosphorylation. Different yeast metabolic states can be induced by simply altering the carbon source in their media. Glucose is the primary carbon source preferred by yeast and induces glycolysis and anerobic fermentation (Otterstedt et al., 2004). In contrast, providing a non-fermentable carbon source, such as glycerol, will switch yeast cells to a respiratory metabolism with oxidative phosphorylation carried out by mitochondria as the major source of ATP (Otterstedt et al., 2004). This metabolic switch also increases ROS levels and arrest or significantly slow down cell division (Otterstedt et al., 2004). All these changes create many untapped opportunities to study the impact on oxidative stress and respiratory metabolism and mitochondrial dysfunction in ALS by simply expressing ALS proteins in cells grown in glycerol (Braun et al., 2011).

A surprisingly understudied area of ALS research is that of cellular stress responses. It seems plausible that at some point the cells’ quality control arsenal fails in ALS-affected neurons and this ultimately gives way to cell death. As we have previously outlined, there are three distinguished, yet interconnected responses that become activated upon cell stress: the HSR, the antioxidative stress response, and the UPR. Yeast present an excellent platform to study cellular stress responses in a quick and effective manner. In fact, the tools to do so have already been optimized and used with great success in yeast (Jonikas et al., 2009; Brandman et al., 2012). Reporter constructs for each response have been developed and expressed in yeast that rely simply on the stress response sequence target of each response’s respective transcriptional activator (Jonikas et al., 2009; Brandman et al., 2012).

## Author Contributions

Both authors wrote the article.

## Conflict of Interest Statement

The authors declare that the research was conducted in the absence of any commercial or financial relationships that could be construed as a potential conflict of interest.
